# Detection of Airborne Nanoparticles through Enhanced Light Scattering Images

**DOI:** 10.3390/s22052038

**Published:** 2022-03-05

**Authors:** Yan Ye, Qisheng Ou, Weiqi Chen, Qingfeng Cao, Dong-Bin Kwak, Thomas Kuehn, David Y. H. Pui

**Affiliations:** 1 Particle Technology Lab, University of Minnesota, Minneapolis, MN 55455, USA; qou@umn.edu (Q.O.); chen3564@umn.edu (W.C.); caoxx409@umn.edu (Q.C.); kwak0068@umn.edu (D.-B.K.); kuehn001@umn.edu (T.K.); dyhpui@umn.edu (D.Y.H.P.); 2 Y2Y Technology, Santa Clara, CA 95052, USA

**Keywords:** airborne nanoparticles, light scattering, light scattering image, aerosol, laser particle detector, image processing

## Abstract

A new method is proposed in this paper to detect airborne nanoparticles, detecting the light scattering caused by both the particle and the surrounding molecules, which can surpass the limitations of conventional laser optical methods while maintaining simplicity and cost-effectiveness. This method is derived from a mathematical analysis that describes the particle light scattering phenomenon more exactly by including the influence of light scattered from surrounding gas molecules. The analysis shows that it is often too much of a simplification to consider only light scattering from the detected nanoparticle, because light scattering from the surrounding gas molecules, whether visible or invisible to the sensor, is important for nanoparticle detection. An image detection approach utilizing the light scattering from surrounding air molecules is described for the detection of airborne nanoparticles. Tests using monodisperse nanoparticles confirm that airborne particles of around 50 nm in size can even be detected using a low-cost testing device. This shows further that even when using a simple image processing code, captured particle light scattering images can be converted digitally into instantaneous particle counts or concentrations. The factors limiting conventional pulse detection are further discussed. This new method utilizes a simple static light scattering (SLS) approach to enable the development of new devices with better detection capabilities, paving the way for the further development of nanoparticle detection technology.

## 1. Introduction

Airborne nanoparticles are of great importance due to their impact on a range of issues, from air pollution to disease transmission [1]. They can cause different adverse effects compared to larger particles due to their high specific surface area and high concentration [2]. Airborne nanoparticles can easily spread over a large area for extended periods and can easily enter and transfer within organisms and interact with cells and subcellular components [3,4].

Detecting particles suspended in the air using optical detection techniques has its own challenges compared to detecting particles suspended in liquids such as in water. Particles in a suspending media can be detected by measuring fluctuations in the intensity of light scattered from moving particles, as in dynamic light scattering (DLS) measurement [5]. This is because when particles move randomly in Brownian motion (motion caused by diffusion only), the diffusivity of suspended particles can be deduced from the autocorrelation function describing the fluctuation signals. For particles suspended in a liquid, it is easy to maintain the motion of particles as Brownian motion, especially when the liquid is confined in a small container or in a stationary droplet. Since 1996, DLS measurements have been widely used, as an ISO standard, to measure the size distribution of particles suspended in liquids, from a few nanometers to about 1 micron [6]. Nanoparticles suspended in a liquid can even be observed under an immersion optical microscope [7] because they can effectively remain in a small optic focus space. For particles suspended in the air, the detection is still challenging. It is not practical to confine air samples in small spaces or small containers or to control the motion of the airborne particles so that the motion is caused only by their diffusion. Since airborne nanoparticles are more mobile and more prone to uncontrolled non-Brownian motion than nanoparticles suspended in liquids, techniques that can successfully detect nanoparticles in liquids, such as DLS or advanced optic microscopes, are rarely used for detecting or analyzing airborne nanoparticles [8,9].

Particles suspended in the air can be detected by measuring the temporal or spatial average intensity of light scattered from particles, as a static light scattering (SLS) measurement [5]. Unlike DLS measurements, which require sufficiently fast samplings, SLS measurements use wide-angle or slow samplings to smooth out the fluctuation in light intensity caused by wave phase differences. SLS measurements are simpler than DLS measurements. However, SLS measurements are limited by the detectable light scattering intensity, which is inversely proportional to the sixth power of the particle diameter. Currently, advanced laser particle counters provided by the world’s leading manufacturers have a lower size detection limit of 100 nm for airborne particles if no growth mechanism is employed to increase the particle size [10]. The particle size growth mechanism increases the complexity of the detection system and limits its applications. An optical detection method that provides nanoparticle detection capability without relying on dynamic light scattering detection or particle growth mechanisms is in high demand, especially for applications hindered by the complexity and compatibility of existing techniques.

This paper presents a new strategy for detecting nanoparticles beyond the detection limit of conventional laser particle detectors. It first introduces an aerosol light scattering equation that includes light scattered from a suspended nanoparticle and from air molecules surrounding the nanoparticle. The equation reveals the effect of light scattered from air molecules, indicating a different path to enhance nanoparticle detection. It then shows the evaluation of a low-cost testing device, which is built for the particle light scattering image detection suggested by the mathematical analysis, using two types of monodisperse particles. The experimental result confirms the achievement of superior nanoparticle detection capability using the new particle detection method. It further demonstrates that the light scattering images of airborne nanoparticles captured in particle detection videos can be further converted to the particle concentration using a simple image processing code, showing that the results of the light scattering image detection method can be presented in the same way as those of the conventional light scattering pulse detection method.

## 2. Theoretical Description

Light scattering from a single nanoparticle can be described well using the Rayleigh equation. For polarized light of intensity$\;I_{0}$ irradiating a nanoparticle, the intensity of the scattered light in the direction of a detector with the angle φ between the oscillating direction of the incident light wave and the direction of the detector can be expressed as:

(1) $$I_{p}=I_{0}\frac{\pi^{2}\;\alpha_{p}{}^{2}}{r^{2}\lambda^{4}}sin^{2}\varphi$$

where λ is the wavelength of the incident light, $\alpha_{p}$ is the polarizability of the particle, and r is the distance between the particle and the detector. When using unpolarized light, the equation is similar–$—sin^{2}\varphi$ is replaced with $\frac{1+cos^{2}\theta }{2}$, where θ is the angle between the direction of incident light and the direction of the detector.

**Figure 1:**
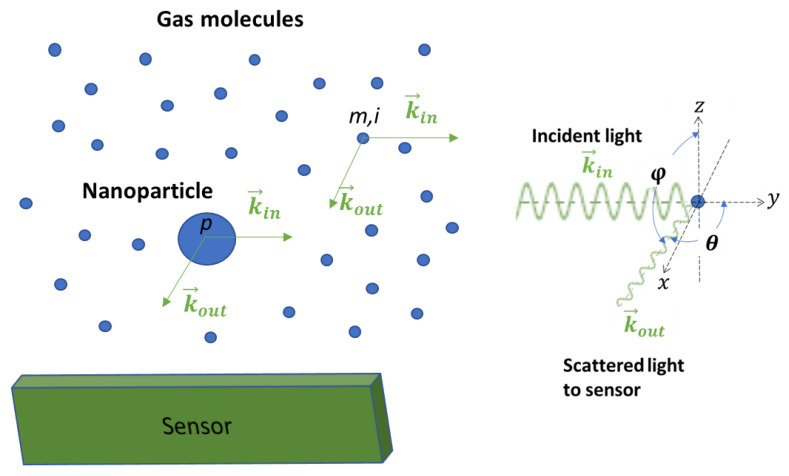
Light scattering when a laser beam irradiates a nanoparticle suspended in the air. Unlike a particle in a vacuum, there are air molecules surrounding the particle, and the effect of light scattering from air molecules should be included in a light scattering model. Using an image detection approach, light scattered from air molecules can be utilized to detect nanoparticles.

Equation (1) describes the situation when there are no gas molecules around the particle, such as in a vacuum. In reality, gas molecules surround the nanoparticle. Figure 1 shows a skitch plot of the light scattering phenomenon when an incident laser beam irradiates a nanoparticle suspended in the air. When a light wave irradiates both the nanoparticle and the surrounding molecules, the electric charges in the nanoparticle and the molecules respond to the changes in the electric field of the light wave, resulting in a collection of oscillating dipoles. These oscillating dipoles emit electromagnetic waves at the same frequency as the light wave, similarly to scattering light from both the nanoparticle and the molecules. Assuming that *n* gas molecules surround a nanoparticle, the detector senses a collection of waves, not just the light wave from the nanoparticle, as shown in Figure 1. The perceived intensity $I_{\;p,nM}$ can be expressed as [11]:

(2) $$I_{\;p,nM}=I_{p}+I_{nM}+2I_{p}\overset{n}{\underset{i=1}{\sum }}\frac{A_{S,i}}{A_{p}}\;cos\;\left(\varphi_{m,i}-\varphi_{p}\right)\;$$

where $I_{nM}$ is the intensity of light scattered from the n gas molecules surrounding the particle and $\varphi_{p}$ and $\varphi_{m,i}$ describe the phases of light waves scattered by the nanoparticle and the gas molecules due to the wave pathlengths.

Equation (2) is the aerosol light scattering equation for particles much smaller than the wavelength of the incident light. Because light scattering is a linear phenomenon and all scattered light is of the same frequency as the incident light, the waves from the particle and molecules exhibit superposition. In the region close to the nanoparticle, where $\phi_{p}\sim \phi_{m,i}$, the interference is constructive, and the last term in the equation is positive [11]. The aerosol light scattering Equation (2) is applicable for both polarized and unpolarized light sources.

Equations (1) and (2) provide valuable particle detection strategies on how to ensure that the scattered light’s intensity is above a sensor sensitivity threshold. For example, if molecular light scattering is not considered, as described in Equation (1), the intensity $I_{p}$ can be increased above a sensor threshold by locating the sensor and the nanoparticle as close as possible to each other. This is because the intensity is inversely proportional to the square of the distance r between the sensor and the particle. This detection strategy has been widely used in conventional approaches, and its limit has already been reached.

Because gas molecules surround a nanoparticle, a detector should sense the light intensity $I_{\;p,nM}$ instead of the light intensity $I_{p}$. The more exact particle light scattering phenomenon described by Equation (2) provides another strategy to ensure that the intensity $I_{\;p,nM}\;$is above the sensor sensitivity, which is to increase the intensity $I_{nM}$ of light scattered from the *n* molecules surrounding the particle. By adjusting the incident light power or sensor settings, the intensity $I_{nM}$ can be used as an adjustable parameter relative to the threshold of the sensor, while the intensity $I_{p}$ can be added to this adjustment because of the superposition principle. For example, the intensity $I_{nM}$ can be adjusted so that $I_{\;p,nM}$ without a particle $I_{\;p,nM}{|}_{I_{p}=0}$ and with a particle $I_{\;p,nM}{|}_{I_{p}\neq 0}$ are all in the detectable regime of the sensor. In this way, the detection of nanoparticles results from comparing the difference between $I_{\;p,nM}{|}_{I_{p}=0}$ and $I_{\;p,nM}{|}_{I_{p}\neq 0}$. Typically, the sensor detection space is much larger than the one-nanoparticle *n*-molecule analysis space used for deriving Equation (2). When using an image-sensing technique, both $I_{\;p,nM}{|}_{I_{p}=0}$ and $I_{\;p,nM}{|}_{I_{p}\neq 0}$ can be observed simultaneously. The molecule light scattering image of $I_{\;p,nM}{|}_{I_{p}=0}$ can be displayed as a background for the particle light scattering image of $I_{\;p,nM}{|}_{I_{p}\neq 0}$, so that the particle can be displayed as a brighter dot image with the molecule light scattering background.

## 3. Materials and Methods

**Figure 2:**
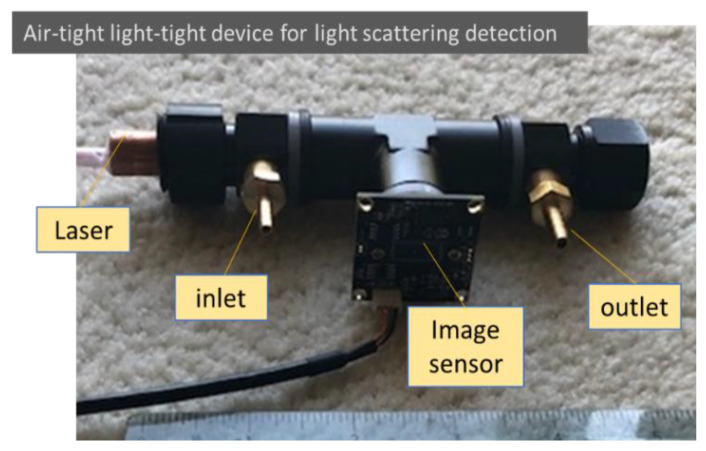
An air-light light-tight device for detecting particle and air molecule light scattering. Low-cost components are used intentionally in the testing setup to check the feasibility for future low-cost applications.

The particle light scattering image detection concept suggested from the mathematical analysis was evaluated using a simple air-tight and light-tight device. The testing device was built using a black anodized aluminum female T-connector with a 3/8-inch NPT thread, as shown in Figure 2. The laser used was a 12 mm compact laser module that could be selected using different wavelengths and powers. The light beam originated on one side and was trapped on the other side of the connector. The image sensor mounted in between was a 2 MP Sony IMX291 image sensor (signal noise ratio: 40 dB; dynamic range: 65 dB) with an M12 lens of 8 mm focal length embedded in the T-connector. An inlet and an outlet allowed air with or without aerosols to be admitted into the observation enclosure. In the tests, the image of light scattering from air molecules was adjusted through the selection of laser power and image sensor sensitivity, as well as image sensor settings, such as gain, exposure, and contrast. The observation volume was determined using the width of the laser beam as well as the observation depth and length.

**Figure 3:**
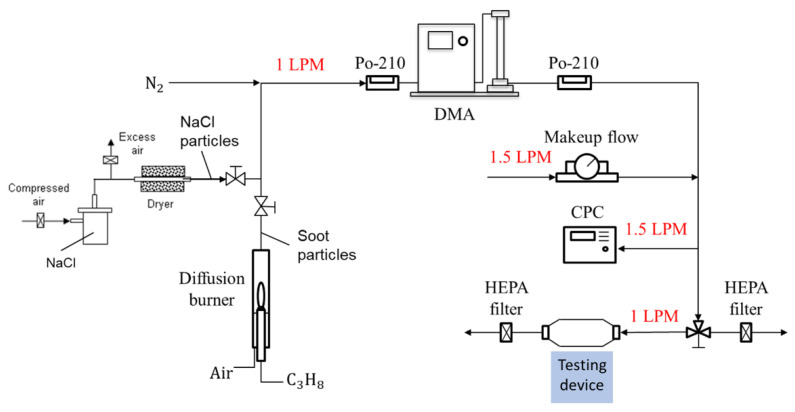
A particle calibration system using monodisperse NaCl and soot particles for evaluating the ability to detect airborne nanoparticles.

A standard particle calibration system was used to generate standard aerosols for checking the detection capability of the detector as shown in Figure 3 [12]. The aerosol particles used for the calibration were NaCl particles generated with a collision-type atomizer and soot particles generated using a home-made diffusion burner, as presented on the left-hand side of Figure 3. NaCl particles served as dielectric particles that were consistent with the conditions required to derive the Rayleigh light scattering equation (Equation (1)) and the aerosol light scattering equation (Equation (2)). NaCl particles are not autofluorescent, and thus, were able to eliminate undesired light emissions, especially when a short wavelength laser was used. Soot particles are typical light-absorbing particles and are a type of air pollutant of research interest. The monodisperse distribution of the particles was achieved by passing them through a charge neutralizer of a radioactive source Po-210 and a differential mobility analyzer (DMA) in the system. After passing through another neutralizer to neutralize the charges on particles exiting the DMA, the particle concentration was checked with a condensation particle counter (CPC), which condensed butanol vapor on the aerosol, with a minimum detection limit of 2 nm. The monodispersed particle was then sampled by the CPC (reference instrument) and the testing device simultaneously, as shown in Figure 2. During the particle detection, the flow through the device was stopped and monodisperse particles were trapped in the detecting space of the testing device. Makeup flows were used to adjust the concentration of particles generated and maintain the DMA and CPC operated under their designed conditions.

## 4. Results

**Figure 4:**
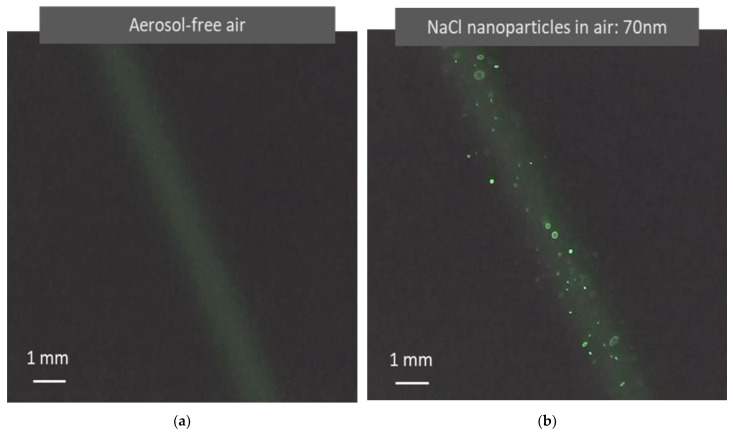
Images of light scattered from air molecules and airborne nanoparticles captured in light scattering detection videos. (**a**) Image formed when a green laser (λ = 532 nm) irradiates aerosol-free air. Unlike the Tyndall effect, the image results from the light scattered from molecules instead of colloidal particles. (**b**) Image formed when a green laser irradiates air containing monodisperse NaCl nanoparticles (70 nm). The nanoparticles become clearly visible as bright dots with air molecule light scattering as a background. The image size difference is caused by optical distortion from the optical system. These images are clearly present when the laser and the image sensor have sufficient power and sensitivity.

Light scattered from air molecules is typically difficult to observe, especially when using a low-power laser with a large beam size. Traditionally, a light beam becomes visible only when colloidal particles are present, which is called the Tyndall effect [13]. In recent years, laser technology and imaging sensing technologies have made tremendous progress, making the observation of light scattered by air molecules much easier than before. Using a simple testing device as shown in Figure 2, light scattered from air molecules is clearly visible in a video recorded with no aerosol, as shown in Figure 4a. In the video, the background scattered light from the air molecules appears relatively uniform. Figure 4b shows a photo from a video recorded when 70 nm monodisperse NaCl aerosol particles are present in the air. The light scattered from the particles shows particle motion against the background of scattered light from the air molecules. This observation agrees with the image superposition described in the aerosol light scattering equation (Equation (2)). In the photo shown in Figure 4b, there is a size difference in the particle light scattering images. By tracking the movement of the light scattering images of individual particles, it is observed that the size difference in the particle scattering images is produced when the particles are located at different distances from the detector.

**Figure 5:**
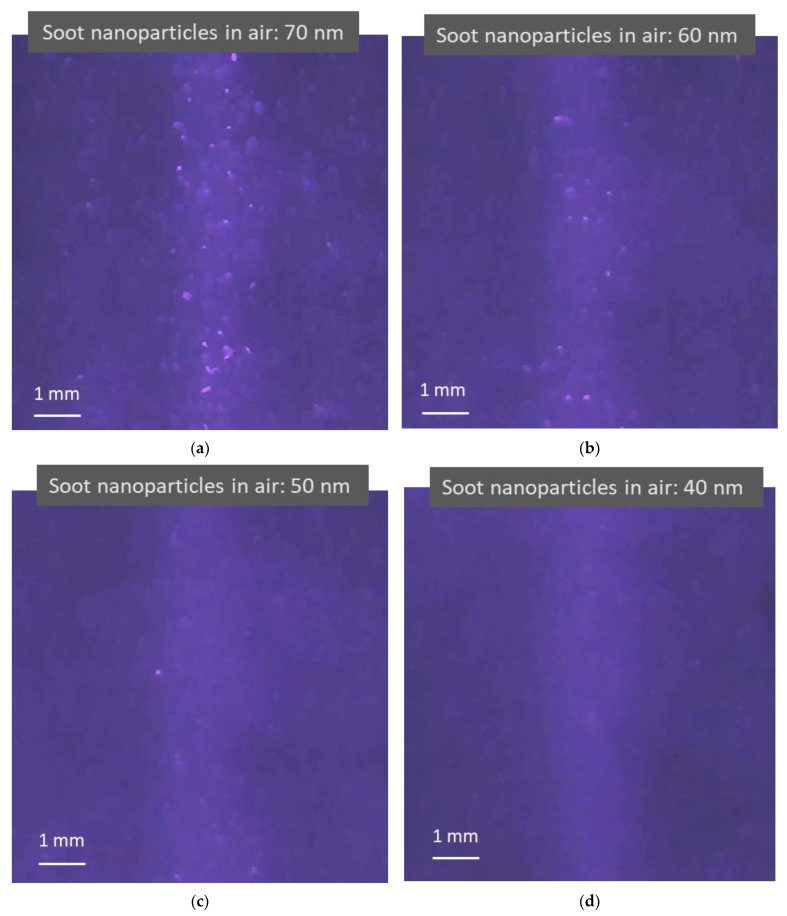
Images of light scattering captured in light scattering detection videos when irradiating air containing light-absorbing soot nanoparticles using a blue laser (λ = 450 nm). (**a**–**d**) Brighter blue images are formed from the particle light scattering of 70, 60, 50, and 40 nm soot nanoparticles and air molecule light scattering, respectively. The lighter blue background appears due to weak light reflections from the testing device surface. As the airborne nanoparticle size decreases, the particle light scattering images change from large bright dots to tiny darker dots mixed with air molecule light scattering.

Figure 5 shows four photos taken from four videos that record light scattering images when four different sizes of monodisperse soot particles are in the air. The concentrations of soot particles used in the test in the figure are 1.7 × 10^5^, 1.3 × 10^5^, 8.8 × 10^4^, and 4.6 × 10^4^ particles per cubic centimeter with a standard deviation of 7.5 × 10^2^, 8.4 × 10^2^, 4.1 × 10^2^, and 3.8 × 10^2^ for 70, 60, 50, and 40 nm soot particles, respectively, as measured using a CNC shown in Figure 3, with an uncertainty of ±5% in the concentration range. As shown in the previous figure, the light scattered from the air molecules is adjusted to be visible to the image sensor. Compared to dielectric NaCl particles, soot particles are light-absorbing particles with a certain electrical conductivity. Images of light scattered from the particles and the background air molecules are still observed. Although there are some distortions in the images, a trend can be observed, in that the smaller the particles, the smaller and darker the particle images.

**Figure 6:**
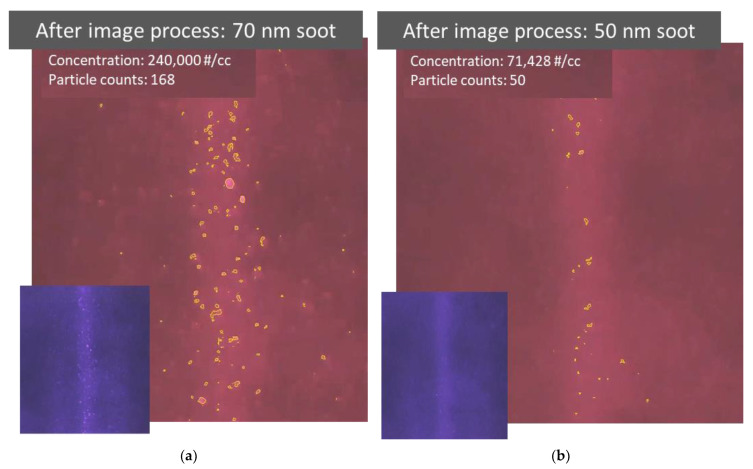
Results after image processing to extract quantitative information from videos recording particle light scattering. (**a**,**b**) Photos taken after image processing of videos recorded while detecting 70 and 50 nm soot particles, respectively. The original video image prior to processing is attached in the bottom left corner. Particles identified by the image processing code are highlighted using a yellow outline. The number count and concentration of identified particles are also provided.

The image information collected in particle detecting videos can be further converted to quantitative information through an image processing code based on a simple contour detection [14]. Figure 6a,b show the results after the quantitative information was extracted from the videos detecting 70 and 50 nm soot particles, respectively. As shown in the photos, the particles identified by the code are highlighted with a yellow outline. The observation volume is determined by the size of the beam and its length in the picture. The count of identified particles in the observation volume and the volume itself are then used to calculate the particle number concentration. The particle count and number concentration are presented near the top-left corner in each processed image. It is quite difficult to see the detected 50 nm particles by eye in the original video. By highlighting the identified particles in the processed video, the detected particles become more visible, as shown in Figure 6b. However, there are some discrepancies between the actual particle concentration and the measured particle concentration calculated using the identified particle counts. For example, the particle concentration converted from the video could be overestimated if the contours of identified particle images are not perfectly described, especially for large particles, or underestimated if the particle identification ratio is low, especially for small particles. The quality of image processing has an important influence on the identification accuracy of particle concentration.

**Figure 7:**
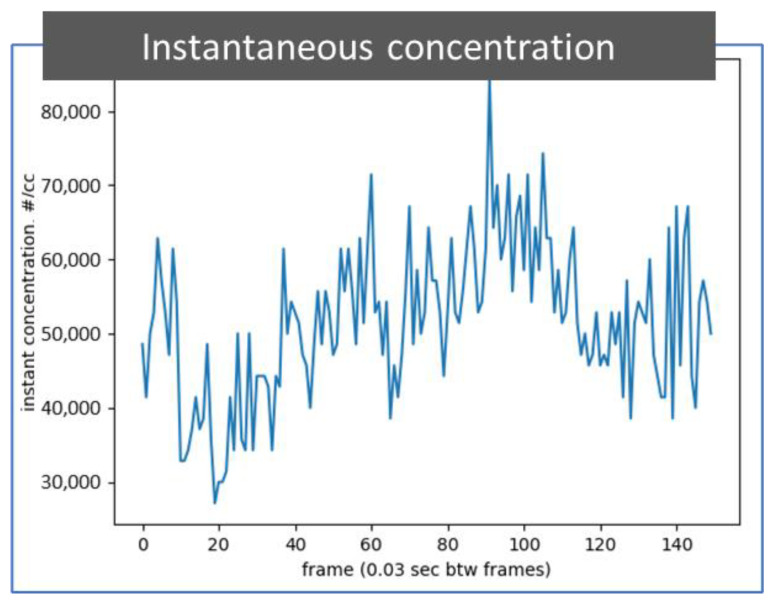
A plot of instantaneous concentration of identified particles. The particle number concentration information is extracted through an image process from 150 photos in a 5 s video while detecting 50 nm soot particles in the air. The particle detection video is the same as presented in Figure 6b. The time between frames is 0.03 s.

Figure 7 depicts a plot that shows the particle concentration of 50 nm soot particles extracted from all 150 photos from a 5-s video recorded at 30 frames per second, which is the same video as that used for Figure 6b. The particle concentration can be considered instantaneous every 0.03 s. The instantaneous particle number concentration is calculated with the count of identified particles in the observation volume in each photo recorded in the particle detection video. This digital particle concentration information can be used to calculate a time average concentration or used to alert the user to or skip an event by comparing the instantaneous concentration with a predetermined value.

## 5. Discussion

In this work, the detection of airborne particles below 100 nm is achieved under the condition that the light scattering from air molecules and nanoparticles is perceivable by the image sensor. Under this condition, the light scattering from airborne nanoparticles can be distinguished from the light scattering from air molecules, utilizing relative motions between nanoparticles and air molecules. The purpose of clearly showing the air molecule light scattering here is to exhibit the superposition characteristics of the light scattering from the nanoparticles and air molecules described in Equation (2). In the actual particle detection process, it is not necessary to obtain the fully visible light scattering of air molecules to detect the nanoparticles, as long as the light intensity difference caused by the particles is perceivable. Because of the superposition, the effect of light scattering from the air molecules exists even when the air molecules’ light scattering is not visible through sensor adjustment. To identify the nanoparticles, it is possible to show only light scattering from particles while hiding the light scattering from air molecules by properly setting the image sensor parameters or the laser intensity.

The superior performance demonstrated in the tests described here raises the question of why conventional approaches based on the detection of light scattering pulses cannot detect aerosols below 100 nm. The light scattering phenomenon in both methods should be the same as described in Equation (2). As estimated from the laser power and beam size in the detection region, the light scattering from air molecules should be sufficiently high to be perceived by a photodetector even in old models of laser particle counters. The main difference is that when using a pulse sensing method, the light waves from the sensor detection space, regardless of whether the light is scattered from a particle or from air molecules, are detected together as a single signal, and a filter is set to distinguish a particle signal from other signals. The high intensity of light scattering from molecules may be undesirable, as the sensor may need to use the filter to remove it as noise, which hurts the sensitivity of the sensor and limits the detection capability. In addition, the pulse sensing method must pass particles through the light beam one after another at a certain speed, which shortens the particle exposure time and increases the difficulty of detecting small particles.

The method described here shows that a light scattering image sensing method can detect aerosols smaller than conventional light scattering pulse sensing methods, but the minimum aerosol size that can be detected remains undetermined. Answering this question requires a better understanding of the physics behind the detection method, in addition to more experiments with sophisticated optics. This may include a better understanding of how large the one-nanoparticle *n*-molecule analysis space in Equation (2) should be, whether the analysis space should be determined based on light wavelength or sensor pixel size, what effect the random motion between the particle and surrounding molecules has, and so on.

This work shows that particles captured in particle detection videos can be expressed as particle counts or particle concentrations, even using an image processing code based on a simple contour detection. It demonstrates that it is feasible to convert particle light scattering image detection results into those using conventional particle light scattering pules detection methods. Certainly, more advanced image processing methods, such as active contours driven by region-scalable fitting and optimized Laplacian of Gaussian energy for image segmentation [15], active contours driven by adaptive functions and fuzzy c-means energy for fast image segmentation [16], and a level-set method based on additive bias correction for image segmentation [17], can be used to make the conversion more accurate and efficient.

The image sensing method described here is unique compared to conventional methods as well as some novel nanoparticle detection methods, such as those based on high-Q whispering gallery micro-lasers [18] or resonant cantilevers [19]. In addition to the ability to detect nanoparticles, the new method provides additional detection capabilities, such as a large detection space, multiple particle detection, and visualization. The detection device is simple and compact. Even when using low-cost readily available components in the testing device, the performance of detecting particles smaller than 100 nm is demonstrated. This method is already attractive for many applications before being improved further. It opens up a new avenue for a variety of detectors, from sophisticated instruments to personal or wearable devices, for many new applications, from monitoring outdoor or indoor air pollution to detecting viruses and toxic nanomaterials.

## 6. Conclusions

The air molecules surrounding suspended particles respond to changes in the electric field of the illuminating light wave, emitting light waves as light scattering in the same way as the suspended particles. The light scattered from the air molecules surrounding airborne nanoparticles should not be ignored or filtered out when detecting the suspended nanoparticles. As shown by the mathematical analysis described herein, the light scattering from air molecules surrounding particles can be utilized for detecting nanoparticles, especially when using a light scattering image detection. When a light scattering image detecting method is used, the particle detection becomes light intensity difference detection, instead of the light scattering pulse detection used in conventional methods, enabling breakthroughs in nanoparticle detection performance. The experimental result using monodisperse particles shows that particles smaller than 100 nm can be detected through simple static light scattering detection. The nanoparticle detection performance can even be achieved using a simple optical system with low-cost components. When further using an image processing algorithm, the particle light scattering images recorded in a particle detection video can be further presented as other forms of digital information of detected particles, such as instantaneous particle count and concentration. Even though further improvements are needed and there are many unknowns to be further addressed experimentally and theoretically, this method is already attractive for many applications. As lasers and image sensors become better, less expensive, and more compact, the method outlined here will become more powerful and cost-effective for detecting airborne nanoparticles.
